# Mental health management in the context of TB care in the WHO European Region: a consensus statement

**DOI:** 10.5588/ijtldopen.25.0661

**Published:** 2026-05-11

**Authors:** C. Vilaplana, O. Konstantynovska, O. Rzhepishevska, L. Codecasa, L. Larocca, D. Zaçe, B. Häcker, R. Otto-Knapp, R. Duarte, M. Pinheiro, A. Aguiar, M. Janković, I.A. Pérez-Hernández, J.P. Millet, I. Nordstoga, M. Drage, A.M. Dyrhol-Riise, A. Dudnyk, P. Fraisse, M. Fréchet-Jachym, F.-X. Blanc, Z. Nanovic, G. Günther, A. Eichenberger, S. Degtyareva, J. Callum, M. Dedicoat, A. Dumitrescu, J. Bruchfeld, G. Auguste, D. Aznar, A. Lacoma, J. Cuevas, J. de Pablo

**Affiliations:** 1 Experimental Tuberculosis Unit, Germans Trias i Pujol Research Institute and Hospital (IGTP), Badalona, Spain;; 2 Department of Genetics and Microbiology, Universtiat Autònoma de Barcelona, Cerdanyola del Vallès, Spain;; 3 Centro de Investigación Biomédica en Red de Enfermedades Respiratorias (CIBERES), Madrid, Spain; 4 Department of Infectious Diseases and Clinical Immunology, V. N. Karazin Kharkiv National University, Kharkiv, Ukraine;; 5 Regional Phtisiopulmonological Center of the Kharkiv Regional Council, Kharkiv, Ukraine;; 6 Department of Chemistry, Umeå University, Umeå, Sweden;; 7 Department of Clinical Microbiology, Umeå University, Umeå, Sweden;; 8 SSD Tisiologia Clinica E Preventiva, Centro Regionale di Riferimento per il Controllo della Tubercolosi, Istituto Villa Marelli- ASST Grande Ospedale Metropolitano Niguarda, Milano, Italy;; 9 Unit of Infectious Diseases, Department of Clinical and Experimental Medicine, ARNAS Garibaldi Nesima Hospital, University of Catania, Catania, Italy;; 10 Department of Systems Medicine, University of Rome Tor Vergata, Rome, Italy;; 11 DZK – German Central Committee Against Tuberculosis, Berlin, Germany;; 12 EPIUnit, Instituto de Saúde Pública, Universidade do Porto, Porto, Portugal;; 13 Instituto de Ciências Biomédicas Abel Salazar (ICBAS), Universidade do Porto, Porto, Portugal;; 14 Instituto de Saúde Pública Doutor Ricardo Jorge, Porto, Portugal;; 15 Public Health Unit Barcelos/Esposende, Barcelos/Esposende Health Local Unit, Universidade do Porto, Porto, Portugal;; 16 Departamento de Ciências Químicas, Faculdade Farmácia, Universidade do Porto, Porto, Portugal;; 17 School of Medicine, University of Zagreb, Zagreb, Croatia;; 18 Department for Respiratory Diseases, University Hospital Centre Zagreb, Zagreb, Croatia;; 19 Unidad de Enfermedades Infecciosas, IBIMA-Plataforma BIONAND, Hospital Universitario Virgen de la Victoria, Málaga, Spain;; 20 Epidemiology Service, Public Halth Agency of Barcelona, TB Center Serveis Clinics, and CIBER de Epidemiología y Salud Pública (CIBERESP), Barcelona, Spain;; 21 LHL International Tuberculosis Foundation, Oslo, Norway;; 22 Department of Infectious Diseases, Oslo University Hospital, Oslo, Norway;; 23 University of Oslo, Oslo, Norway;; 24 Innovation in Respiratory Infections and Tuberculosis Diagnosis Group, Institut d’Investigació Germans Trias i Pujol (IGTP), Barcelona, Spain;; 25 Department of Tuberculosis, Clinical Immunology and Allergy, National Pirogov Memorial Medical University, Vinnytsia, Ukraine;; 26 Réseau National des Centres de lutte Antituberculeuse and the GREPI Working Group of the Société de Pneumologie de Langue Française (SPLF), Paris, France;; 27 Centre Hospitalier de Bligny, Briis-sous-Forges, France;; 28 Nantes Université, CHU Nantes, Service de Pneumologie, L’institut du Thorax, Nantes, France;; 29 The GREPI Working Ggroup of the Société de Pneumologie de Langue Française (SPLF), Paris, France;; 30 Institute for Lung Diseases and Tuberculosis, Skopje, Republic of North Macedonia;; 31 Faculty of Medical Sciences, Goce Delcev University, Stip, Republic of North Macedonia;; 32 Department of Pulmonology, Allergology and Clinical Immunology, Inselspital, Bern, Switzerland;; 33 University Hospital, University of Bern, Bern, Switzerland;; 34 Department of Infectious Diseases, Inselspital, Bern University Hospital, University of Bern, Bern, Switzerland;; 35 Krankenhaus St. Raphael Ostercappeln, Germany;; 36 Homerton University Hospital NHS Foundation Trust, NHS England, University of Sydney, Sydney, NSW, Australia;; 37 Sydney Medical School, University of Sydney, Sydney, NSW, Australia;; 38 Department of Infection, University Hospitals Birmingham, Birmingham, UK;; 39 Marius Nasta Institute of Pneumology, German Centre for Infection Research (DZIF), Eastern European Study Site, Bucharest, Romania;; 40 Unit of Infectious Diseases, Department of Medicine Karolinska Institute, Department of Infectious Diseases, Karolinska University Hospital, Stockholm, Sweden;; 41 TB Center Serveis Clinics, Barcelona, Spain;; 42 Department of Psychiatry, Germans Trias i Pujol Research Institute and Hospital and Universitat Autònoma de Barcelona, Catalonia, Spain.

**Keywords:** tuberculosis, mental disorders, stigma, integrated care, WHO guidelines, drug-resistant TB

## Abstract

**BACKGROUND:**

Integrated care for TB and mental health is critical, as mental disorders worsen TB outcomes. WHO guidelines recommend routine screening for mental health conditions and integrated care for TB patients, yet implementation remains uncertain.

**METHODS:**

We conducted a survey with the participation of 34 experts from 26 countries from the WHO European Region within TBnet and the ADVANCE-TB COST Action, followed by a Delphi process to assess the uptake of WHO recommendations and build consensus on strategies for integrated TB–mental health care.

**RESULTS:**

The survey showed substantial gaps: only 21% of experts, representing six countries, reported local or national guidelines integrating mental health into TB care, and systematic screening was rare, including for drug-resistant TB. Building on these findings, the Delphi process reached consensus on the need for validated screening tools at key treatment stages, structured referral pathways, and tighter alignment between TB and mental health services. Eighteen consensus recommendations were developed, addressing training, routine data collection, technology-supported care, and sustainable financing.

**CONCLUSION:**

Mental health remains insufficiently integrated into TB care across the WHO European Region. Routine screening coordinated referral systems and implementation of the proposed recommendations are essential to deliver people-centred, equitable, and effective TB care.

Mental disorders, including substance use disorders, increase TB incidence and worsen outcomes by affecting well-being, health care–seeking behaviour, and treatment adherence.^1–4^ TB is also more common among people with mental illness and those with substance use disorders,^5^ particularly among poor and marginalised populations, and many TB survivors experience impaired mental health.^6^ Additionally, TB can trigger anxiety and depression through stigma, social isolation, and treatment-related burdens, including catastrophic costs.^7^ Integrated care for TB and mental health, alongside investigation of underlying drivers, is therefore essential. According to a recent WHO recommendation, all TB patients should be assessed for mental health conditions – as per the International Classification of Diseases 11th Revision (ICD-11) definition, syndromes characterised by clinically significant disturbance in an individual’s cognition, emotional regulation, or behaviour reflecting dysfunction in the psychological, biological, or developmental processes that underlie mental and behavioural functioning – or substance use disorders at treatment initiation and routinely thereafter, using validated screening tools to identify and manage issues.^3^ Comprehensive protocols and interventions for implementation in non-specialised health settings have been published by the WHO in 2008.^8^ However, in our opinion and experience, these recommendations were not being systematically implemented within TB care. Based on this, we hypothesised that mental health issues during TB diagnosis and treatment are underdiagnosed, inadequately managed, and underreported, with existing guidelines in the WHO European Region not sufficiently implemented. Our aim was to map gaps in reporting and managing concurrent TB and mental health issues, identify improvement opportunities, and propose recommendations developed under Delphi consensus methodology.

## METHODS

The study examined existing laws and guidelines for managing mental health issues during TB diagnosis, programmatic screening and management of mental health conditions in TB patients, access to TB and psychiatric/psychological treatment, and the existence of public registries documenting TB cases with mental health comorbidities. This collaborative effort between the TBnet (https://www.tbnet.eu) and ADVANCE-TB COST Action (https://www.advancetb.eu) consortia was conducted in three phases.

### Phase 1 (November 2023–February 2024)

A survey was distributed via email to members of both consortia (Supplementary Data S1), primarily TB clinicians, researchers, and public health practitioners in WHO European Region countries. No formal ethics approval was sought as the study did not involve patients or sensitive personal data. All panellists provided informed consent. Only name, institutional affiliation, and email address were collected, and responses were anonymised. The study complies with the General Data Protection Regulation (Regulation EU 2016/679). The protocol was reviewed within the ADVANCE-TB and TBnet consortia, confirming ethics approval was unnecessary. Of the 53 WHO European Region Member States, 26 (49%) participated in this first phase (Supplementary Data Table S1).

### Phase 2 (March–August 2024)

Phase 1 participants were invited to join the Task Force. Alongside TB clinicians, researchers, and public health practitioners, mental health specialists were incorporated to support interpretation. In total, 32 stakeholders from 14 countries accepted and helped shape country-level responses (Supplementary Data Table S2). Task Force experts compiled a structured document describing their national context, supported technically to ensure consistency and completeness. Country documents followed an eight-domain template covering aspects of the mental health–TB intersection: 1) existing guidelines; 2) programmatic screening and management of mental health conditions in individuals with TB; 3) associated gender-related risks; 4) programmatic TB screening for individuals with mental disorders; 5) access, cost, and availability of TB treatment; 6) access to mental health services and links with TB care; 7) existence of registries capturing TB cases with mental health comorbidities; and 8) proposed statements for inclusion in the Delphi process. National experts also identified relevant published studies through targeted searches (primarily PubMed) and existing knowledge; this was not systematic and served only to provide contextual examples. These inputs were synthesised to identify key characteristics and recurring themes, informing the draft recommendation statements.

### Phase 3 (September 2024–March 2025)

The Task Force developed recommendation statements using an online Delphi process. The core research team drafted initial statements following strategy discussions at the 2024 TBnet Annual Meeting and 2024 European Respiratory Society Congress, with refinement based on Task Force input. Two anonymous survey rounds were conducted, without a formal external methodologist. Consensus was defined as ≥70% agreement or disagreement (scores 5–7 or 1–3 on a 7-point Likert scale). Although no formal GRADE review was undertaken, each recommendation was assigned a Strength of Consensus: strong (≥90% agreement) or moderate (70%–89%). No additional consensus meeting was held.

We collaboratively drafted the manuscript and used ChatGPT (OpenAI, version 5) to improve its readability and clarity. The authors reviewed and edited the text and assume full responsibility for the final content.

## RESULTS

### Phase 1 survey results

We collected data from 34 experts representing 26 WHO European Region countries (Supplementary Data Table S2). Participants included health care professionals in pulmonology, infectious diseases, public health, microbiology, psychology, and psychiatry, as well as representatives from civil society and non-governmental organisations (NGOs) supporting TB patients. Only 21% of respondents reported guidelines or recommendations for mental health management in the context of TB. Moreover, 44% indicated availability of programmatic screening and/or management of mental health conditions for TB patients, while 32% reported systematic TB screening for individuals with mental disorders. Regarding gender-related risks, 41% of experts noted gender patterns in comorbid TB and mental health conditions. Several experts observed that alcohol and drug use among TB patients are more prevalent among men, often linked to war-related trauma or the challenges faced by unaccompanied migrant minors, while diagnosed mental health disorders – particularly depression – were more frequent among women with TB.

### Analysis at the country level: existing legal directives, guidelines, and recommendations for managing mental health issues in the context of TB diagnosis and treatment

Guidelines or directives addressing mental health management in the context of TB were identified in 8 of the 14 countries analysed (Table 1). The table reflects only countries represented in the Task Force, with national experts supplementing survey responses through review of local and national guidelines; no data are presented for countries without representation. France, Germany, Italy, Norway, Romania, Sweden, Switzerland, and Ukraine have guideline recommendations, typically generic or focused on specific populations. Spain and the Russian Federation lack comprehensive governmental guidance, although partial recommendations exist. Mental health and substance use disorders complicate treatment tolerance, adherence, and outcomes in TB patients.^4^ Countries with guidelines vary in approach. In France and Germany, guidelines recommend translators, assessment of social risk factors such as socio-economic and legal status, and the evaluation for psychiatric diseases. In France, a directive^9^ states that asylum seekers’ morbidity mainly results from physical and psychological violence, thus recommending prioritisation of psychotrauma treatment, mapping of care services for vulnerable populations, referrals to psychiatric services, training for private practitioners, and the availability of 10 psychotrauma care facilities across the country. In Germany, post-traumatic stress disorder and other psychiatric diseases are screened for, especially for drug-resistant TB (DR-TB) cases, and corresponding interventions are offered. However, management is typically on an individual basis with limited systematic guidance despite WHO and countries such as Switzerland^10^ integrating mental health in TB guidelines. Italy and Norway include psychosocial support in national TB guidelines. The Italian National Institute for Health, Migration and Poverty promotes bio-pyscho-social interventions for vulnerable populations that respect cultural values,^11^ although specific mental health directives remain generic.^12^ In Norway, the non-profit organisation Landsforeningen for hjerte, lunge og hjerneslag (LHL) has indicated that guidelines in the ‘Psychosocial Follow-Up’ section^13^ are inconsistently implemented across most hospitals and depend on resources and staff interest. Romania’s 2022 TB guidelines acknowledge the need for psychological support, but national health insurance covers only post-COVID-19 conditions, limiting access despite a separate 2020 guideline for mental health screening in patients with DR-TB.^14^

**Table 1.** Summary of the status of mental health management in the context of TB across various WHO European Region countries.

Country	Guidelines/directives	Key points	Challenges
Croatia	No specific guidelines or recommendations	No local or regional initiatives addressing mental health in TB; TB incidence considered low	Declining TB incidence leading to perception of low public health concern
France	Directive for health care pathways for newly arrived migrants	Emphasis on psychotrauma treatment; map medical and medico-social structures; refer to mainstream services; train practitioners; professional interpreting services	Implementation consistency; ensuring access to trained professionals and interpreting services
Germany	Guidelines recommendations; WHO guidelines integrated into national TB guidelines	Systematic approach recommended; evaluation for PTSD, depression; offer psychosocial support and treatment for comorbidities	Insufficient financial support dedicated for socio-medical care; language barriers; stigma
Italy	Ministry of Health guidelines; NIHMP guidelines; WHO and ERS guidelines	Holistic patient care includes mental health; psychosocial support for migrants; bio-psychosocial interventions considering cultural values	Recommendations are broad and lack detailed practical considerations
Republic of North Macedonia	General guidelines by Ministry of Health; no specific TB-related mental health guidelines	General psychiatric treatment guidelines	Lack of TB-specific mental health guidelines
Norway	National TB guidelines include ‘Psychosocial Follow-Up’ section	Focus on psychosocial follow-up; implementation depends on resources and health care worker knowledge	Inconsistent implementation across hospitals
Portugal	No specific laws or guidelines; delayed implementation of National Mental Health Plan	No mention of mental health in TB surveillance reports; lack of scientific recommendations	Structural and organisational service issues; lack of professional education and local/regional initiatives addressing mental health in TB
Romania	National TB guideline (2022); separate guideline (2020) for DR-TB	Screening for mental health conditions in DR-TB patients; PHQ-4 scale for assessment; referral needed for psychiatric consultations	Health insurance does not cover psychological services for pulmonary diseases except post-COVID-19 conditions
Russian Federation	Order of the Ministry of Health (Minzdrav) of Russia from 15.11.2012 N 932н	Positions for psychiatrist-narcologist and psychotherapist recommended; focus on psychological support	Funding issues; shortage of qualified specialists; inconsistent use of psychological support models
Spain	Literature-based recommendations; no formal governmental guidelines	Directly observed therapy for patients with mental health conditions; patient-centred approach; systematic mental health screening	Lack of formal recommendations and systematic mental health support
Sweden	National recommendations	Recommendations include having TB-trained nurses screen for mental health disorders related to anti-TB medications; migrants are screened for both TB and mental health disorders.	No educational materials are available in foreign languages; challenges arise when patients are contagious, violent, or refuse anti-TB antibiotics.
Switzerland	TB guidelines integrating mental health	Focus on psychiatric assessment and Directly Observed Treatment when needed. Newly arrived migrants and asylum seekers are offered TB and mental health screening at federal asylum-seeking homes.	Absence of formal screening programmes
United Kingdom	General management under the Mental Health Act; no specific TB-related mental health guidelines	Management of mental health issues governed by general guidelines	Absence of TB-specific mental health guidelines
Ukraine	National health care standards for TB	Integrated services for TB patients using illicit drugs; family-centred approach; active drug safety monitoring; psychiatric counselling for mental disorders	Shortage of psychiatrists and psychologists; varying levels of implementation across regions

PTSD = post-traumatic stress disorder; NIHMP = National Institute for Health, Migration and Poverty; ERS = European Respiratory Society; DR-TB = drug-resistant TB; PHQ-4 = Patient Health Questionnaire-4.

Other countries focus on specific themes of TB management. Switzerland focuses on psychiatric assessment and Directly Observed Treatment when needed, while Sweden’s 2022 guidelines^15^ mention mental health only regarding TB drug side effects. However, in practice, legal framework^16^ and medical staff engagement allow addressing mental health conditions in patients with TB. In Ukraine, TB standards emphasise integrated mental health, particularly for patients using illicit drugs^17^ requiring psychiatric referral before TB treatment. Active drug safety monitoring includes adverse event screening and psychiatric counselling, though specialist shortages hinder implementation.

According to Task Force members, several countries lack TB-specific mental health guidelines or recommendations. In Portugal, the National Mental Health Plan has been systematically delayed, and the latest 2022 TB surveillance report omits mental health,^18^ with no recommendations from scientific groups on this matter. In Croatia, the absence of local or regional initiatives likely reflects the declining incidence of TB^19^ and the perception that it is not a significant public health concern. The UK includes no TB-specific guidance in the Mental Health Act.^20^ In the Republic of North Macedonia, psychiatric guidelines issued by the Ministry of Health do not specifically address mental health disorders in the context of TB.^21^ In the Russian Federation, TB care providers are required to include positions for psychiatrist-narcologists and psychotherapists.^22^ However, this does not guarantee their presence due to lack of funding and shortage of qualified specialists, although some researchers are addressing this issue by developing models and principles for psychological support in TB patients.^23^ In Spain, no formal Ministry of Health guideline was identified; however, published scientific literature provides relevant guidance, emphasising directly observed and patient-centred approaches,^24,25^ as well as the need for systematic mental health screening and referral.^26^

### On programmatic screening and/or management of mental health conditions for individuals with TB at the country level

In the WHO European Region, mental health screening and management for individuals with TB are generally undertaken by the treating clinician and supported by national public health services, with referral to mental health specialists when required. This relies on existing mental health infrastructure rather than integrated TB–mental health programmes. Routine or systematic mental health screening among TB patients is uncommon, except in Spain, France, Germany, Austria, and Norway. In Spain, screening targets specific groups such as people living with HIV and individuals who use intravenous drugs,^27^ while France^28^ and Switzerland focus on newly arrived migrants and asylum seekers. In Switzerland, comprehensive questionnaires are administered at federal asylum-seeking homes which include items on TB symptoms and mental health concerns despite the absence of formal screening programmes. Norway’s guidelines recommend systematic psychosocial screening throughout treatment using tools like the Hopkins Symptom Checklist-10, but implementation is limited by time, resources, interpreter shortages, and reliance on clinical intuition rather than formal screening tools. In Sweden, infectious diseases staff also manage TB in people with psychiatric comorbidities, particularly among refugees, though challenges arise with non-compliant patients. Even with legally allowed isolation, current solutions are suboptimal and staff receive limited training for such cases. Germany, Italy, and Ukraine recommend screening (especially in DR-TB),^29^ but adherence is unclear. In some settings, screening and support are delivered outside national TB programme structures, rather than as routine services. For example, in Romania, externally funded projects have provided social and psychological support, while in Italy, pilot programmes in regions like Lombardy and Lazio integrate TB and mental health care through multidisciplinary teams and NGOs.^30^

We have identified several common barriers for implementing screening and care management of concomitant TB and mental health disorders (Supplementary Data Figure S1). The stigma associated with both TB and mental health disorders can deter patients from seeking help.^31^ In the Russian Federation, psychiatric registration deters care due to legal/employment repercussions. Another barrier is the fragmentation of health care systems, since integrating mental health screening into routine TB care varies across countries and regions due to inconsistent protocols and resource availability, a situation worsened by a lack of vacancies for professionals – especially psychologists – as in Portugal and Spain.^32^ Changing settings also pose challenges: in countries with low TB incidence, such as Croatia,^19^ limited investment in TB control contrasts with rising migration from high-TB-burden countries. This shift highlights the urgent need for legal frameworks and programmatic approaches to ensure effective management in vulnerable and often stigmatised populations.

### Programmatic screening activities of TB for people with mental health disorders

Systematic policies for screening comorbidities remain rare across the WHO European Region. Only France, Spain, UK, Ukraine, North Macedonia, and the Russian Federation conduct programmatic screening, generally limited to specific populations in most countries. In France, psychiatric patients and people deprived of their liberty are screened by TB centres (Réseau National des Centres de Lutte Antituberculeuse).^33,34^ In Spain, the National Health Service responsibilities belong to the Regional Governments. In Catalonia, the Public Health Agency screens individuals attending drug-dependence centres, although implementation is limited across the region.^35^ Similarly, in the UK, TB screening occurs only in certain settings such as substance abuse clinics, not nationwide. In Switzerland, asylum seekers are offered TB and mental health screening at federal centres and referred if considered appropriate. Italy does not classify mental illness as a TB risk factor; thus, screening is not mandated.^11^ In Ukraine, all suspected mental disorder cases should be screened,^17^ but ongoing war severely disrupts implementation, especially near front-line regions. Additionally, psychoneurological nursing houses require patients to be free of infectious diseases, necessitating TB exclusion. The Republic of North Macedonia recognises mental disorders and alcohol abuse as TB risk factors with annual preventive programmes coordinated by the Institute for Lung Diseases and Tuberculosis.^36^ In the Russian Federation, psychiatric patients must undergo biannual TB X-ray screening,^22^ although adherence is compromised by stigma and fear of social or employment repercussions.

### Costs and accessibility to TB and mental health management

In most countries, TB management costs are fully or partly covered by national health systems. In France, treatment is free via social security or schemes for undocumented migrants.^33^ Italy, Spain, Portugal, and the UK cover diagnosis and care, with TB drugs provided through public services which purchase them at Ministry-approved prices,^37^ either universally or for vulnerable groups. The Republic of North Macedonia covers costs through the Ministry of Health, while in Ukraine, the Global Fund partially reimburses phthisiopulmonology centres. In Germany and Switzerland, mandatory health insurance covers TB diagnosis, treatment, hospital stays, and ambulatory care, while refugees are supported by social services or migration authorities.^38^ In the Russian Federation, TB treatment is primarily funded through regional budgets with state support and, to a lesser extent, insurance funds.^39^ At a European level, drug access is hindered by demand fluctuations and procurement delays, with previous studies documenting treatment costs and drug availability.^40,41^ Hospitalisation adds fixed costs to therapy,^42^ while high prices and delays in obtaining DR-TB drugs remain major concerns.^43^ Furthermore, social costs of TB,^44^ geographic and transport barriers, workforce shortages, and long distances to treatment centres further increase patient and health care expenses.

Psychiatric and psychological care is available in all countries analysed with varying accessibility. In Spain, Italy, France, Croatia, the UK, Norway, and the Russian Federation, services are state-funded; in Germany and Switzerland, covered by mandatory health insurance; and in Romania, only for insured. In Ukraine, regional psychoneurological hospitals include an infectious disease department, with some wards for TB patients. Yet, formal links between TB and mental health care are often missing in some countries such as Croatia, France, Spain, Italy, and Norway. Hospital-based psychiatric consultancies exist, but outpatient psychological care is harder to access, especially in rural areas or for migrants.^45^ Community initiatives support refugees and asylum seekers, but access remains limited^38^ despite higher need.^46^ The unclear impact of TB on access to mental health care underscores the need for systematic approaches and comprehensive recommendations to address service and access barriers for TB patients with mental health issues.

Although equal access to mental health care is recognised as a civil right in most countries analysed, accessing these services remains challenging. Public systems prioritise severe disorders, leaving mild cases with insufficient support.^47,48^ Long waiting lists, staff shortages, and budget cuts force patients to seek costly private care,^49,50^ delaying diagnosis and treatment. In Ukraine in 2019, 15% of TB patients reported out-of-pocket expenses for psychological care.^51^ Additionally, stigma, inadequate regulation, and lack of interest in mental health issues further restrict access, despite the known impact of mental illness on TB vulnerability and treatment outcomes.

### Data collection and registry of TB cases with concomitant mental health disorders

The UK, North Macedonia, and Ukraine are the only countries with registries that include concomitant mental health disorders. In the UK, mental health conditions are recorded as a separate risk factor, including impact on patient’s ability to self-administer treatment, with data summarised annually in the UKHSA annual TB report.^52^ Access to these databases is restricted. In the Republic of North Macedonia, data protection aligns closely with the EU General Data Protection Regulation.^53^ Italy maintains separate TB and mental health registries but lacks an integrated database. In Spain, regional TB registries exist, but only aggregated annual reports are published annually by the National Centre for Epidemiology and Public Health Surveillance, without details on mental health comorbidities. Some scientific articles have incorporated this data,^26^ but databases remain publicly inaccessible. In Germany, the Robert Koch Institute publishes TB reports based on local public health data, again without mental health comorbidities, as is also the case in Italy, Portugal, and the Russian Federation. Nonetheless, a German national project (No1Lost) aims to develop a DR-TB registry including socio-economic and mental health data. Table 2 presents the Task Force’s identified advantages and barriers to integrating data on mental health comorbidities into TB registries.

**Table 2.** Advantages and barriers of integrating data on mental health comorbidities into existing or new TB databases/registries.

Advantages	Barriers
Improved Patient Care: Enhanced monitoring and follow-up for TB patients with mental health comorbidities.	Supranational Coverage: Ideally, the database should operate at an EU level for complete data collection.
Valuable Data: Collection of reliable data on the prevalence and incidence of TB with accompanying mental health disorders.	Public Accessibility: The database should be generally accessible to support research and interventions, with some controlled access.
Research Enablement: Facilitation of studies exploring the interplay between TB and mental health conditions.	Ethical Issues: Must address stigma and ensure informed consent for health data use.
Data Homogenisation: Standardisation of data across regions to ensure quality and consistency.	Data Protection: Need strict compliance with GDPR and other relevant laws.
Policy Development: Support for targeted interventions and public health strategies based on robust evidence.	Technical Challenges: Include building data infrastructure, selecting CRFs, using open-source tools like REDCap, and securing funding.
	Promotion: Expansion requires support from public entities, government bodies, WHO, scientific societies, and consortia like ADVANCE-TB or TBnet.
	Physician Participation: Strategies are needed to motivate physicians’ involvement.
	Financial Considerations: Evaluate cost-effectiveness, especially given Europe’s relatively low TB incidence.

ADVANCE-TB: research network, funded by the COST (European Cooperation in Science and Technology) (CA21164); CRF = Case Report Form; GDPR = General Data Protection Regulation from the European Union; TBnet = European grass-root consortium, https://www.tbnet.eu/.

### Experts’ recommendations

Best practices for addressing mental health in routine TB care were identified by the Task Force (Supplementary Data Figure S2). During Phase 2, national experts also reported examples of published studies from their countries that included mental health data in the context of TB (Supplementary Data Table S3). These contextual examples were intended to aid interpretation rather than serve as analytical findings. Following the Phase 2 country-level analysis, an initial set of recommendation statements was developed and submitted to a two-round online Delphi process (Figure). This resulted in 18 final consensus recommendations (Table 3), intended for adaptation to local contexts.

**Figure.**
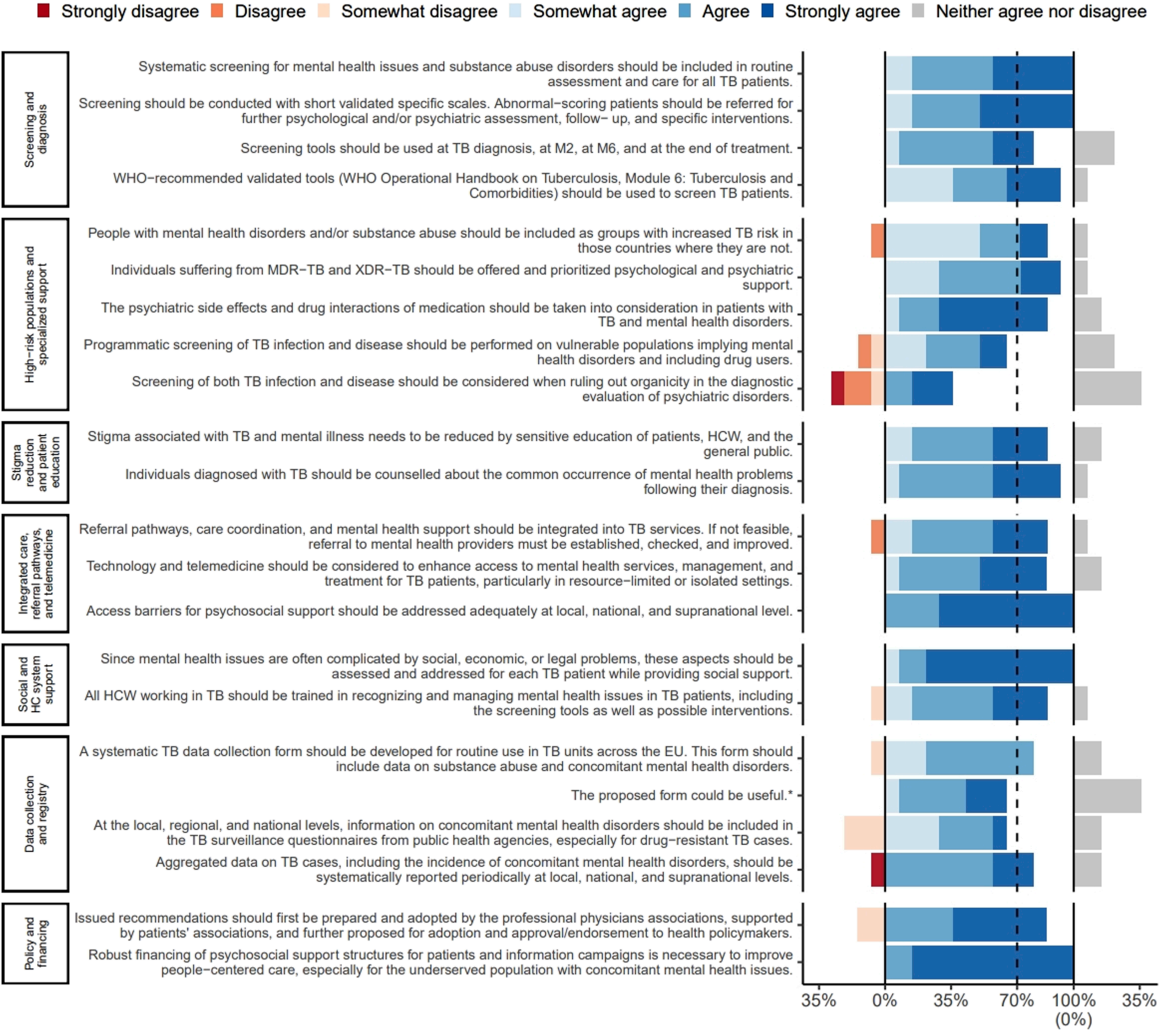
Agreement reached for each statement in the Delphi consensus. The figure illustrates the percentage of consensus reached for each thematic area assessed during the Delphi process. The vertical axis refers to key areas of consensus and the horizontal axis to level of agreement in percentage (%). A consensus of 70% agreement was required to include a recommendation. MDR-TB = multidrug-resistant TB; M2 = month 2 after starting treatment; M6 = month 6 after starting treatment; WHO = World Health Organization; XDR-TB = extensively drug-resistant TB. *Form presented as Supplementary Data S2.

**Table 3.** List of recommendations based on the statements that reached a consensus.

Recommendations		Strength of consensus
#1	A systematic screening for mental health issues and substance abuse disorders should be included in routine assessment and care for all TB patients.	Strong
#2	The screening should be conducted with short validated specific scales and, if scoring abnormally, they should be referred for further psychological and/or psychiatric assessment, follow-up, and specific interventions.	Strong
#3	Mental health screening for TB patients should be conducted at diagnosis, at month 2 (M2), at month 6 (M6), and at the end of treatment (if not coinciding with M6), in accordance with WHO guidelines for monitoring changes in symptoms over time.	Moderate
#4	There is consensus that short, validated screening tools should be used for TB patients. We propose employing the WHO-recommended tools (WHO Operational Handbook on Tuberculosis, Module 6: Tuberculosis and Comorbidities – Mental Health Conditions, Geneva: WHO; 2023): the Patient Health Questionnaire-9 (PHQ-9) for depression, the Generalised Anxiety Disorder Assessment-7 (GAD-7) for anxiety, assessments for psychosis symptoms, and the Alcohol Use Disorders Identification Test (AUDIT) and/or the Alcohol, Smoking and Substance Involvement Screening Test (ASSIST) for substance use disorders.	Strong
#5	People with mental health disorders and/or substance abuse should be recognised as high-risk groups for TB in countries where they are not currently included.	Moderate
#6	Individuals with multidrug-resistant TB and extensively drug-resistant TB represent populations with a high prevalence of mental illness. Psychological and psychiatric support should therefore be prioritised and made readily available for these patients.	Strong
#7	The psychiatric effects of anti-TB drugs should not be overlooked. When managing patients with both TB and mental health conditions, the use of antidepressants, anxiolytics, antipsychotics, and anti-TB medications must consider potential side effects and drug interactions.	Moderate
#8	Efforts to reduce the stigma associated with TB and mental illness should include sensitive education for patients, health care workers, and the general public.	Moderate
#9	Individuals diagnosed with TB should receive counselling on the common occurrence of mental health issues following diagnosis to enable early symptom recognition and timely help-seeking. Additionally, psychoeducation should be provided to those who do not require specialised care, equipping them with self-help strategies for managing their symptoms.	Strong
#10	Mental health support should be integrated into TB services via established referral pathways and coordinated care. Where direct integration is not feasible due to logistical challenges, limited resources, or cost-effectiveness (as may occur in very low-TB-incidence countries with decentralised care), robust referral pathways to mental health providers must be established, monitored, and improved. In addition, peer-to-peer advice can serve as an effective interim measure to facilitate psychological support for TB patients. Local-level barriers to accessing psychosocial support should be adequately addressed.	Moderate
#11	Technology and telemedicine should be leveraged to enhance access to mental health services, management, and treatment for TB patients, especially in resource-limited or isolated settings.	Moderate
#12	Access barriers for psychosocial support must be adequately addressed at the local, national, and supranational levels.	Strong
#13	Given that mental health issues are often compounded by social, economic, or legal challenges, these factors should be assessed and addressed for each TB patient. Tailored social support should be provided by engaging the social sector, public health agencies, and local communities.	Strong
#14	All health care workers involved in TB care should receive training to recognise and manage mental health issues. This training should include the proper use of screening tools, knowledge of available interventions (e.g., brief interventions, substance withdrawal management, and supportive structures), and strategies to avoid stigmatising language and practices related to both TB and mental health conditions.	Moderate
#15	A systematic TB data collection form should be developed for routine use in TB units across the EU. This form must include information on substance abuse and concomitant mental health disorders, whether pre-existing or developed during TB disease or treatment.	Moderate
#16	Aggregated data on TB cases, including the incidence of concomitant mental health disorders, should be systematically reported at local, national, and supranational levels. Such data should be disaggregated by biological sex and gender.	Moderate
#17	Recommendations should first be adopted by professional physicians’ associations, supported by patient associations, and then proposed for adoption, approval, and endorsement by health policy makers (e.g., the Ministry of Health).	Moderate
#18	Robust financing for psychosocial support structures and information campaigns is essential to improve people-centred care, particularly for underserved populations with concomitant mental health issues.	Strong

Strength of consensus is defined as follows: strong = ≥90% agreement; moderate = 70%–89% agreement.

## DISCUSSION

Our findings highlight a substantial gap in guideline formulation and implementation at the TB–mental health intersection across the WHO European Region. Policies are often fragmented, generic, or inconsistently implemented, leaving many patients without systematic psychosocial support. This gap underscores the urgent need to integrate mental health into TB care.

Survey responses suggested that gender-related patterns may influence comorbid mental health conditions in TB. Experts reported that alcohol and drug use were more common among men, sometimes linked to trauma or social vulnerability, whereas depression and other diagnosed mental disorders were reported more frequently among women. These observations highlight the potential relevance of gender when designing mental health support within TB care. Although mental health is acknowledged in many TB guidelines, implementation remains inconsistent due to workforce shortages, limited funding, language barriers for migrants, and stigma. In low-incidence settings, limited political and financial attention constrains integrated care, while universal screening in such settings would likely be inefficient. Addressing these barriers requires system-level approaches rather than relying on individual clinician initiative. Such approaches should integrate TB and mental health specialists, social workers, and communities, while being supported by sustainable financing and aligned with national health strategies.

Systematic data collection is crucial, yet few countries include mental health variables in TB registries, limiting surveillance, evaluation, and policy design. A specialised TB database at EU, national, or local levels could enhance patient care, research, and public health, though privacy, ethical, and funding challenges remain. In the short term, adding mental health fields to existing TB surveillance questionnaires could capture key data on risk factors, comorbidities, and substance use, despite practical barriers (Supplementary Data Table S3). Gender-related risks^31,54–58^ also highlight the need for sex-disaggregated data, though European evidence remains scarce.

Stigma reduction must be central to policy. Interventions should combine patient-centred care, awareness campaigns, and professional training to normalise mental health support in TB treatment. Despite challenges like migration, conflict, and inequality, integrating mental health into TB programmes is essential for clinical care, equity, human rights, and health system resilience.

This study has several limitations. Our expert panel covered 26 of the 53 WHO European Region countries, which may limit generalisability. Countries without representation were not included, and as guideline availability does not guarantee dissemination or implementation, this remains an area requiring setting-specific strategies and further evaluation. Furthermore, the panel initially comprised mainly TB professionals; four mental health experts were added in Phase 2 to ensure appropriate clinical input, but broader representation could further strengthen future analyses. Also, limited evidence prevented full use of GRADE methodology, which could be considered if more systematic reviews on TB–mental health integration emerge.

Our consensus statement highlights the urgent need to integrate mental health and substance use screening into TB care using brief, validated tools at key treatment points for early detection and referral. Recognising these disorders as major TB risk factors – especially in DR-TB cases – underscores the importance of psychological and psychiatric support. Integrated services require robust referral pathways, comprehensive health care worker training, and technology and telemedicine to overcome access barriers. Reducing stigma, providing tailored social support, and systematic collection of mental health data are also essential. Finally, collaboration among professional associations, patient groups, and policymakers, supported by sustainable financing, ensures people-centred care and improved TB outcomes. Collectively, these recommendations provide a roadmap to enhance TB treatment outcomes and overall patient well-being across diverse settings.

## Supplementary Material
