# Supplementary material for: Mental health management in the context of TB care in the WHO European Region: a consensus statement
Source: IJTLD Open. 2026 May 11;3(5):275–85. doi: 10.5588/ijtldopen.25.0661 (PMC13160271; doi:10.5588/ijtldopen.25.0661)
Supplement: Supplementary file 1 [file ijtldopen25-0661_supplementarydata1.pdf]

Supplementary Table S1. Countries included in this study. TB burden is defined as per WHO criteria as follows: low – less than 10 cases per 100 000 population, lower-moderate – between 10 and 49 cases per 100 000 population, upper-moderate – between 50 and 99 cases per 100 000 population, endemic – between 100 and 299 cases per 100 000 population. From World Health Organization (2024). Tuberculosis incidence (per 100 000 population). Available at https://data.who.int/indicators/i/13B4226/C288D13 (accessed July 23, 2025) and World Health Organization (2021). WHO global lists of high burden countries for TB, multidrug/rifampicin-resistant TB (MDR/RR-TB) and TB/HIV, 2021–2025. Licence: CC BY-NCSA 3.0 IGO.

Country	TB incidence per 100 000 population (2023)	TB burden	Study phase
Armenia	25	Lower-moderate	Phase I
Belgium	7.5	Low	Phase I
Croatia	3.5	Low	Phase I and II
Denmark	3.6	Low	Phase I
Estonia	8.2	Low	Phase I
Finland	3.4	Low	Phase I
France	8.3	Low	Phase I and II
Georgia	55	Upper-moderate	Phase I
Germany	4.8	Low	Phase I and II
Hungary	5.9	Low	Phase I
Ireland	4.6	Low	Phase I
Italy	4.4	Low	Phase I and II
Latvia	16	Medium	Phase I
Lithuania	28	Medium	Phase I
Republic of North Macedonia	10	Lower-moderate	Phase I and II
Poland	10	Lower-moderate	Phase I
Portugal	16	Lower-moderate	Phase I and II
Romania	55	Upper-moderate	Phase I and II
Russian Federation	38	Lower-moderate	Phase I
Slovakia	4.3	Low	Phase I
Spain	5.9	Low	Phase I and II
Sweden	3.7	Low	Phase I and II
Switzerland	5.4	Low	Phase I and II
Turkey	13	Lower-moderate	Phase I
United Kingdom	7.6	Low	Phase I and II
Ukraine	112	Endemic	Phase I and II

Supplementary Table S2. Task Force Members: Expertise and Country Representation for Evaluation of WHO Guidelines on Mental Health in TB Care (EU Context). Members are listed in alphabetical order.

Member	Primary Role / Expertise	Country Represented (Data Reported)
Aguiar, Ana	TB Public Health Researcher / Community Outreach	Portugal
Auguste, Gaston	TB Mental Health Expert / Psychosocial Care Provider	Spain
Blanc, François-Xavier	Clinical TB Expert	France
Bruchfeld, Judith	Clinical TB Expert	Sweden
Callum, Jack	Clinical TB Expert	United Kingdom
Codecasa, Luigi	Clinical TB Expert	Italy
Cuevas, Jorge	Clinical Mental Health Expert with Management Role	Spain
Dedicoat, Martin	Clinical TB Expert	United Kingdom
Degtyareva, Svetlana	Clinical TB Expert	Russian Federation
de Pablo, Joan	Clinical Mental Health Expert with Management Role	Spain
Drage, Mona	NGO Representative / Advocacy Expert	Norway
Duarte, Raquel	Clinical TB Expert	Portugal
Dudnyk, Andrii	Clinical TB Expert	Spain
Dumitrescu, Andreea	TB Mental Health Expert / Psychosocial Care Provider	Romania
Dyrhol-Riise, Anne Ma	Clinical TB Expert	Norway
Eichenberger, Anna	Clinical TB Expert	Switzerland
Fraisse, Philippe	Clinical TB Expert	France
Fréchet-Jachym, Mathilde	Clinical TB Expert	France
Günther, Gunar	Clinical TB Expert	Switzerland
Häcker, Brit	TB Policy / Institutional Representative	Germany
Janković, Mateja	Clinical TB Expert	Croatia
Konstantynovska, Olha	Clinical TB Expert	Ukraine
Larocca, Licia	Clinical TB Expert	Italy
Millet, Joan Pau	Clinical TB Expert	Spain
Nanovic, Zorica	Clinical TB Expert	Republic of North Macedonia
Nordstoga, Ingunn	NGO Representative / Advocacy Expert	Norway
Otto-Knapp, Ralf	TB Policy / Institutional Representative	Germany
Pérez-Hernández, Isabel Ascensión	Clinical TB Expert	Spain
Pinheiro, Marina	TB Clinical Researcher	Portugal
Rzhepishevska, Olena	Preclinical TB Researcher	Sweden
Vilaplana, Cristina	TB Clinical Researcher & TB Program Coordinator	Spain
Zaçe, Drieda	Clinical TB Expert	Italy

Supplementary Table S3. Summary of European published TB studies including mental health data

Country	Reference	Year	Population	Results
Spain	Romero-Tamarit et al.[1]	2017	Longitudinal cohort of individuals with TB followed up in the Metropolitan area of Barcelona	12.7% of TB patients with concomitant psychiatric disorders in the cohort. People aged 41–60 years, born in Spain, and those with a psychiatric disease history were found to be at high risk of mental health impairment with the questionnaire used (K-10) at BL; with a subsequent early improvement throughout the follow-up period.
Spain	Casas et al.[2]	2024	People included for DOT in a reference centre for managing and treating vulnerable people with TB in Catalonia. TB referential centre in Catalonia for hard to reach populations, complex TB cases or individuals with TB who have socio-economic or health issues that interfere with treatment adherence.	32.2% of people included for DOT shown to o have one or several mental health disorders, and that this was associated with loss to follow-up. Mental health disorders and behavioural disorders were associated with poor adherence to TB treatment.
Portugal	Ramos et al.[3]	2023	People with or affected by TB, including individuals who are directly suffering from TB and those who are indirectly impacted by the disease due to social, cultural, and economic factors. The article highlights various barriers these individuals face, such as low health literacy, stigma, discrimination, socioeconomic vulnerabilities, and gender-related inequalities, which can all affect treatment outcomes by impeding access to proper care and hindering treatment adherence.	Impact on Treatment Adherence: Mental health issues are identified as a significant barrier to treatment adherence. Addressing mental health through psychoeducation, psychological first aid, and timely interventions is crucial for improving treatment outcomes.
Portugal	Sousa et al.[4]	2024	Individuals with TB who also experience mental health disorders. The article specifically examines the prevalence and impact of mental disorders, such as depression and anxiety, among TB patients, including those with MDR.	Mental health disorders are much more common among TB patients, especially those with MDR-TB. These issues often lead to poor treatment adherence, increasing the risk of treatment failure, prolonged infectiousness, and drug resistance. TB and mental health have a bidirectional relationship: TB can cause mental health problems due to stress, stigma, and isolation, while existing mental disorders can raise TB risk through weakened immunity and unhealthy coping strategies. Social factors like poverty, unemployment, and lack of support further worsen the situation. Additionally, many healthcare systems lack the resources to integrate mental health services into TB care,

				negatively impacting patient outcomes. To address these challenges, routine mental health screening, integrated care models, and stronger social support systems are recommended.
Croatia	Zmak et al.[5]	2017	35 patients (in the period from 2007-2014) had TB caused by a single Mtb strain and the focal point of infection was one regional psychiatric hospital.	Description of a TB outbreak among patients with mental health disorders in Croatia. The outbreak emphasized the vulnerability of mentally ill patients to TB and the infection control measures complexity in psychiatric institutions but it also showed the consequence of low TB awareness in this setting.
Russian Federation	Mordyk et al.[6]	2016	42 patients with HIV/TB treated in the Omsk clinical TB dispensary and consent to participate	Low commitment to therapy due to mood swings and a long therapy duration is revealed. The attitude to the disease was characterised by demonstration of low awareness about the disease and belief of patients in treatment results, in the absence of any personal actions. The conclusion is made that for those patients, psychological work is necessary and psychological and social support of treatment is crucial. An individualised approach is important.
Russian Federation	Zolotova et al.[7]	2019	318 pulmonary tuberculosis patients aged 18-60 years old who underwent in-patient treatment in Central Tuberculosis Research Institute in 2017-2019,	A comparative study of the psychological characteristics of pulmonary tuberculosis patients demonstrating different levels of therapeutic cooperation allowed identifying psychological prognostic parameters of therapeutic cooperation, the main of which were the severity of suspicion, negative affective states (irritability, aggressiveness), distrustful-sceptical style of interaction, confrontation with others, as well as the quality life, primarily the emotional and social aspects of functioning.
Russian Federation	Zahkarova & Filshtinskaya[8]	2020	800 subjects aged 19–60 years with TB, residents of Samara (Russia).	Of all the TB patients, 68% showed a moderate level of social frustrations; 31% belonged to a group of people with an uncertain assessment of frustrations; and 1% of the respondents had pronounced social frustrations. The TB patients were reported to be most dissatisfied with their physical and material condition, lifestyle in general and professional activities.

BL: baseline; DOT: Directly Observed Treatment; MDR-TB: multidrug-resistant tuberculosis; Mtb: M. tuberculosis.

[1]Romero-Tamarit A et al. A longitudinal prospective study of active tuberculosis in a Western Europe setting: insights and findings. Infection 2024; 52:611–23. https://doi.org/10.1007/S15010-024-02184-2.

[2]Casas X et al. Mental Health Influence on the results of Directly Observed
Tuberculosis Chemotherapy. Mental health and Tuberculosis. Communication at SEPAR’s congress. Valencia, España; 2024

[3]Ramos JP et al. Building bridges: multidisciplinary teams in tuberculosis prevention and care. Breathe 2023; 19: 230092. https://doi.org/10.1021/acsinfecdis.4c00466

[4]Sousa S, Aguiar A. Syndemic Burden: Bridging the gap between Tuberculosis and Mental Health Care for Integrated Patient-Centered Solutions - a comprehensive review. Mental Health: Global Challenges 2024; 7:14–26. https://doi.org/10.56508/mhgcj.v7i1.180

[5]Zmak et al. Neglected disease in mentally ill patients: Major tuberculosis outbreak in a psychiatric hospital. American Journal of Infection Control. 2017 (45), 4:456-457. https://doi.org/10.1016/j.ajic.2016.09.004

[6]Mordyk AV et al. Lichnost patsiyenta TB/HIV. Dalnevostochny Meditsinsky Zhurnal, 2016 (1), 50–53. (In Russ.)

[7]Zolotova NV et al. Specific psychological features of new pulmonary TB patients in the context of psychotherapeutic care during in-patient treatment. TB and Lung Diseases, 2019; 97(1), 18–24. (In Russ.)

[8]Zahkarova EV, Filshtinskaya EG. Psychosocial characteristics of tuberculosis patients in Russia and treatment compliance factors. RUDN Journal of Psychology and Pedagogics 2020; 17:330–47. https://doi.org/10.22363/2313-1683-2020-17-2-330-347

Supplementary Document S1. Questionnaire shared in Phase 1 through the TBnet (https://www.tbnet.eu/) and Advance-TB COST (https://www.advancetb.eu/) Consortia.

Availability of mental health management in the context of TB diagnosis and TB treatment across WHO European Region

There is evidence of bidirectional links between TB and mental health conditions. Our hypothesis is that mental health in both in diagnosis and TB management is underreported, poorly managed and appropriate guidelines are missing in the WHO European Region. The aim of this project is to map the gaps in reporting and managing concomitant mental health issues across individuals with TB in the WHO European Region, to identify the improvement opportunities, and to propose recommendations. By filling out this form you agree that we will process your data in line with our privacy policy.

1. Name, affiliation

____

2. Which country do you represent?

____

3. Are there any laws/guidelines, recommendations in your country regarding how to manage mental health issues in the context of TB diagnosis and during TB treatment?

- o Yes, national
- o Yes, regional
- o Yes, local
- o No

4. If you answered yes to Q3, are these laws/guidelines implemented?

- o Yes, always
- o Sometimes (depending on the regions, hospitals, centres, etc.)
- o Never

5. If you answered no to Q3, do you think it would be useful to have guidelines/would you be interested in such guidelines

- o Yes
- o No

6. There is evidence that people suffering from TB are at risk of mental health impairment. Are there any programmatic screening and/or management of mental health conditions for individuals with TB in your country?

- o Yes, at national level
- o Yes, at regional level
- o Yes, at local level
- o Depends on the centres/hospitals/…?
- o No

7. Could concomitant mental health conditions and TB bear any gender related risks in your country?

- Yes
- No

8. If you answered yes to Q7, please provide details

___

___

9. There is evidence that people suffering from mental health disorders are at risk of TB. Are there in your country any programmatic TB screening activities for people with mental disorders?

- Yes, national
- Yes, regional
- Yes, local
- No

10. Is the TB treatment free in your country? (If you want to add more about it please see next question)

- Yes
- No

11. Please feel free to comment your answer to Q10 if you want to

___

___

12. Is psychiatric and/or psychological treatment free in your country? (If you want to add more about it, please see next question)

- Yes, both
- Yes, psychiatric
- Yes, psychological
- None

13. Please feel free to comment your answer to Q12 if you want to

___

___

14. Is there any public database/registry of the TB cases with concomitant mental health disorders for your country?

- Yes
- No

15. If you are aware of a public database/registry, please provide the link to it

___

16. Please add any information you think might be appropriate

___

___

17. Your e-mail

___

Supplementary Document S2. Form proposed for systematic mental health data collection form for routine TB management and discussed within the Delphi Process. ASSIST: Alcohol, Smoking and Substance Involvement Screening Test; AUDIT: Alcohol Use Disorders Identification Test; GAD-7: General Anxiety Disorder 7 Questionnaire; PHQ-9: Patient Health Questionnaire 9; TB: tuberculosis.

Checklist for systematic mental health data collection form for routine TB management							
1	**Mental health disorder previous to the TB episode?**	Yes	No				
If yes to 1), please provide additional information on which specific mental health disorder.							
**Depression assessment**		**During TB episode:** Time from TB diagnosis (Baseline, Month 2, End of treatment)					
		Baseline		M2		End of treatment	
2	PHQ-9 administered?	Yes	No	Yes	No	Yes	No
If yes to 2), which was the total score obtained? (Score 0-4: None or minimal Depression severity, Score 5-9: Mild, Score 10-14: Moderate, Score 15-19: Moderately severe, 20-27: Severe)							
	Need intervention?	Yes	No	Yes	No	Yes	No
	If intervention needed, specify intervention						
	Need to be referred?	Yes	No	Yes	No	Yes	No
	If referred, specify centre						
**Anxiety assessment**		**Time from TB diagnosis:** (Baseline, Month 2, End of treatment)					
		Baseline		M2		End of treatment	
3	GAD-7 administered?	Yes	No	Yes	No	Yes	No
	If yes to 3), which was the total score obtained? (Score 0-4: Minimal Anxiety, Score 5-9: Mild Anxiety, Score 10-14: Moderate Anxiety, Score greater than 15: Severe Anxiety)						
	Need intervention?	Yes	No	Yes	No	Yes	No
	If intervention needed, specify intervention						
	Need to be referred?	Yes	No	Yes	No	Yes	No
	If referred, specify centre						
**Psychosis assessment**		Baseline		M2		End of treatment	
4	Symptoms of psychosis? (Marked behavioural changes; neglecting usual responsibilities related to work, school, domestic or social activities; Agitated, aggressive behaviour, decreased or increased activity; Fixed false beliefs not shared by others in the person's culture; Hearing voices or seeing things that are not there; Lack of realization that one is having	Yes	No	Yes	No	Yes	No

	mental health problems)						
	If yes to 4), anti-TB medication suspected?	Yes	No	Yes	No	Yes	No
	If yes to 4), anti-TB medication suspended?	Yes	No	Yes	No	Yes	No
	If anti-TB medication suspended, improvement after next 1-2 weeks?	Yes	No	Yes	No	Yes	No
	If any other intervention applied, specify intervention						
	Referred?	Yes	No	Yes	No	Yes	No
	If referred, specify centre						
**Substance abuse assessment**		Baseline		M2		End of treatment	
5	Substance abuse suspected?	Yes	No	Yes	No	Yes	No
6	If yes to 5), AUDIT administered?	Yes	No	Yes	No	Yes	No
	If yes to 6), which was the total score obtained?						
	Need intervention?	Yes	No	Yes	No	Yes	No
	If intervention, specify intervention						
	Need to be referred?	Yes	No	Yes	No	Yes	No
	If referred, specify centre						
7	If yes to 5), ASSIST administered?	Yes	No	Yes	No	Yes	No
	If yes to 6), which was the total score obtained?						
	Need intervention?	Yes	No	Yes	No	Yes	No
	If intervention, specify intervention						
	Need to be referred?	Yes	No	Yes	No	Yes	No
	If referred, specify centre						
**Self-harm & suicide assessment**		Baseline		M2		End of treatment	
8	Self-harm/Suicide ideation?	Yes	No	Yes	No	Yes	No
	Need intervention?	Yes	No	Yes	No	Yes	No
	Specify intervention?						
	Referred?	Yes	No	Yes	No	Yes	No
	If referred, specify centre						

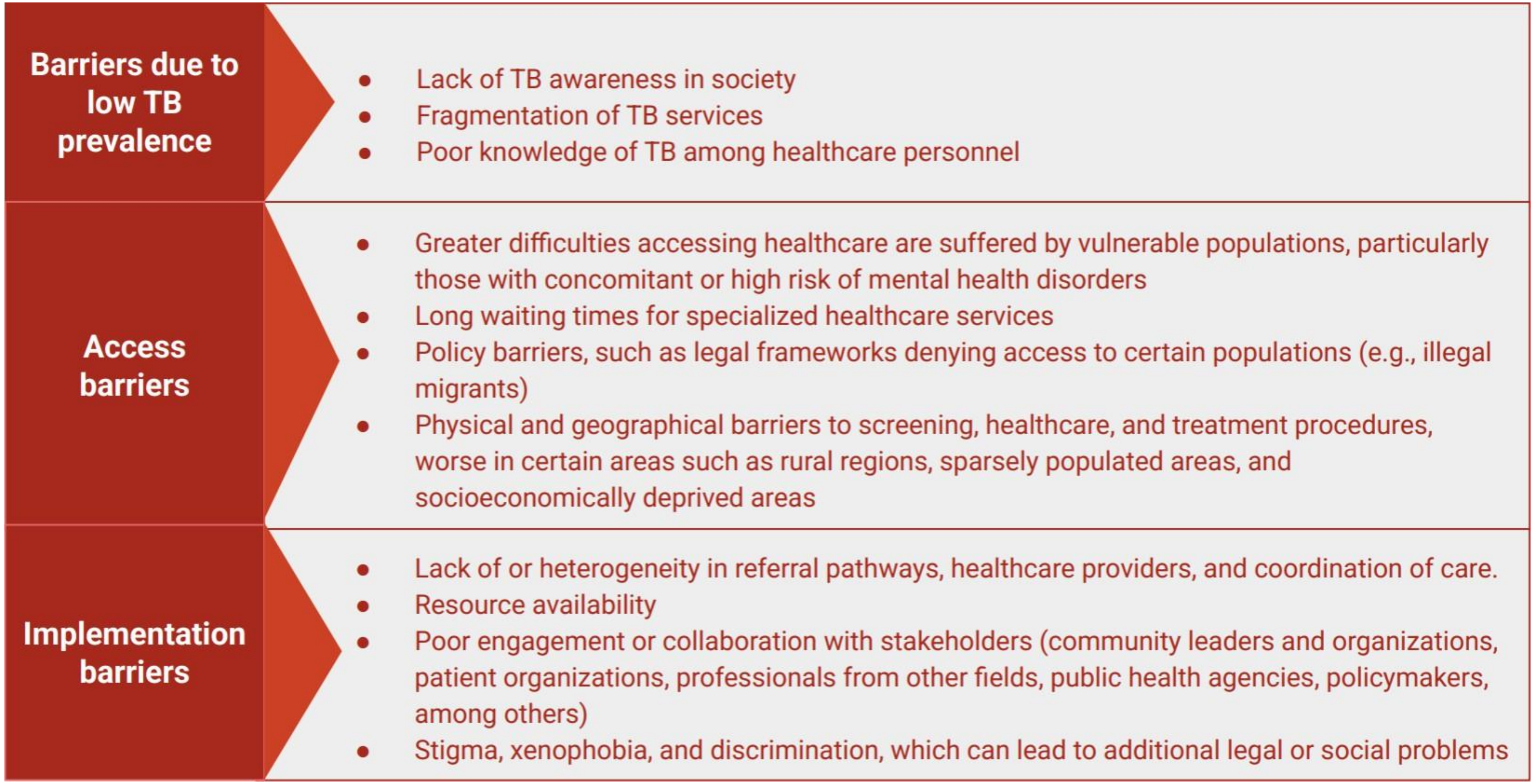

Supplementary Figure S1. Common barriers for implementing screening and care management of concomitant TB and mental health disorders. TB: tuberculosis.

During routine TB healthcare management

Build multidisciplinary teams

Raise awareness and train HCW to recognize and manage mental health issues in individuals with TB and conditions related to substance use, overdose and intoxication; and to avoid the use of stigmatising language and practices related to both TB and mental health conditions.

Implement systematic screening for mental health issues and substance abuse disorders during TB healthcare management at baseline, during treatment, and at the end of treatment:

- Screen for depression using PHQ-9
- Screen for anxiety using GAD-7
- Check for symptoms of psychosis
- Screen for substance abuse using AUDIT and ASSIST

Provide counseling about the common occurrence of mental health problems to all individuals diagnosed with TB

Provide social support and protection if appropriate

If appropriate, consider psychological interventions

If symptoms of distress:
If trained and supervised psychological-intervention providers are available, consider:
mental health conditions WHO Problem Management Plus (PM+); WHO Self-Help Plus (SH+) – a brief guided self-help package
If detecting anxiety:
Rule out pharmacology reason +
Apply interventions including psychoeducation on anxiety, training in stress management skills, prescribing physical exercise, or applying cognitive behavioral therapy brief interventions
If symptoms of psychoses:
Rule out the drug responsible and suspend the suspected anti-TB medication
If substance abuse:
Promote withdrawal or at least stabilizing substance use
→ Check* for care options dependent on the complexity of symptoms and impairment in functioning

If no improvement is observed or considered appropriate, refer to mental health professionals

Ensure pathways for timely and effective referral to mental healthcare providers and professionals, and establish a system for continuous feedback. If these pathways are not in place, they need to be developed. Additionally, all TB unit professionals should identify and be familiar with their designated mental healthcare provider and the referral pathways.

→ Check[&] for models of integrated care for people with tb and comorbidities

Supplementary Figure S2. Recommended practices to address mental health conditions during routine TB healthcare management. ASSIST: Alcohol, Smoking and Substance Involvement Screening Test; AUDIT: Alcohol Use Disorders Identification Test; GAD-7: General Anxiety Disorder 7 Questionnaire; HCW: Health Care Workers; PHQ-9: Patient Health Questionnaire 9; TB: tuberculosis. *: WHO operational handbook on tuberculosis. Module 6: tuberculosis and comorbidities - mental health conditions. Geneva: World Health Organization; 2023. [&]: Framework for collaborative action on tuberculosis and comorbidities. Geneva: World Health Organization; 2022. Licence: CC BY-NC-SA 3.0 IGO.
